# Beyond pleasurable and meaningful: Psychologically rich entertainment experiences

**DOI:** 10.1371/journal.pone.0315596

**Published:** 2025-02-06

**Authors:** Dominique S. Wirz, Allison Eden, Ezgi Ulusoy, Morgan E. Ellithorpe

**Affiliations:** 1 Amsterdam School of Communication Research (ASCoR), University of Amsterdam, Amsterdam, Netherlands; 2 Department of Communication, Michigan State University, East Lansing, Michigan, United States of America; 3 Department of Communication, University of Delaware, Newark, Delaware, United States of America; De Montfort University, UNITED KINGDOM OF GREAT BRITAIN AND NORTHERN IRELAND

## Abstract

Entertainment experiences have been conceptualized as hedonic (pleasurable) or eudaimonic (meaningful), mirroring the hedonic and eudaimonic components of psychological well-being. However, psychologists have proposed a third component of well-being: psychological richness, which is characterized by variety, novelty, and interest. In this paper we explore the role of psychological richness in film and television entertainment experiences. Two studies, an experience sampling study (*n* = 28) and a survey (students in the US, *n* = 247 and general population in Germany, *n* = 289) show the prevalence of experience of psychological richness during media use and its positive relationship with well-being. A replication with a different scale (n = 291) demonstrates that psychologically rich entertainment experiences may have been previously been conflated by some measures of eudaimonic entertainment. Incorporating psychologically rich entertainment experiences as a third addition to hedonic and eudaimonic experiences can increase the intervention potential of media used to enhance well-being.

## Introduction

Well-being refers to optimal psychological experience and functioning [1]. On the one hand, well-being can be considered as primarily hedonic, which refers to the presence of positive affect and the absence of negative affect, or more simply, the experience of happiness in life [2,3]. On the other hand, well-being also may refer to eudaimonia, which refers to a fulfilling and satisfying way of life by actualizing one’s potential, or finding meaning in life [4–6]. Recently, a group of psychologists suggested a third component of well-being, psychological richness, which refers to the variety of interesting and perspective-changing experiences in one’s daily life [7–10]. These researchers argue that leading a happy and meaningful life can be boring at times, and thus individuals seek psychological richness in addition to happiness and meaning [9]. Furthermore, they note that not all individuals are born in circumstances that allow them to live a happy life, and not everyone is interested in searching for meaning in life [8]. Hence, psychological richness can also be considered as a source of well-being for individuals with a lack of happiness and/or meaning in life. Consequently, they promote a tripartite model of well-being, in which hedonia, eudaimonia, and psychological richness are three distinct, but complementary, components of psychological well-being.

Oishi et al. [8] describe that psychological richness can not only be obtained by a broad range of personal experiences, e.g., by travel, but also vicariously through entertainment media such as novels or poetry. This is in line with functional theories of media selection, such as the Uses and Gratifications Approach [11], which states that individuals deliberately select media content to satisfy their psychological needs. Studies have demonstrated that entertainment media use can indeed satisfy hedonic and eudaimonic needs [12,13] and thus positively affect hedonic and eudaimonic well-being after media use [14]. As already presumed by Oishi et al. [8], it seems highly plausible that media use can also satisfy the need for psychological richness by providing a variety of perspectives and vicarious experiences.

Moreover, the experience of psychological richness during media use should contribute to positive appraisal of entertainment media. Although difficult to define, entertainment has often been characterized by noting novelty, variety, and interest [15,16]. Psychologically rich experiences have thus implicitly been included in past descriptions of entertainment experiences. Yet, much research in the past decade has focused on the role of need satisfaction in media enjoyment for hedonic and eudaimonic experiences [12,13]. Hedonic entertainment, or enjoyment, is characterized by experiences of pleasure and comprehension, whereas eudaimonic entertainment, also sometimes called appreciation, is characterized by meaningfulness, moral virtues, or being connected to larger meaning in life or transcendence [17–21]. That said, some authors have conceptualized eudaimonic entertainment in a broader way, that encompasses aspects of psychological richness such as content being thought-provoking [22], boundary expanding [23,24] or challenging [25]. Yet, the unique contribution of thought provoking or interesting content to either hedonic or eudaimonic entertainment experiences, or to subsequent well-being, has not been clearly explicated. We propose to disentangle these concepts and consider psychological richness as a third, distinct type of entertainment experiences, based on the satisfaction of psychological needs for variety, novelty, and interest.

In this research project, we investigate if entertainment experiences can be conceptualized as hedonic, eudaimonic, and psychologically rich and thus make distinct contributions to each of these dimensions of well-being. To do so, we analyze how an individual’s general level of psychological richness in everyday life is related to the experience of hedonic, eudaimonic, and psychologically rich entertainment during media use (RQ1), how entertainment experiences affect feelings of psychological richness after media use (RQ2) and what type of media content is considered to provide psychologically rich entertainment (RQ3). First, we conducted an experience-sampling study with students at a large Midwestern U.S. University (*n* = 28). Participants’ Netflix use was tracked for 10 days, and they reported their entertainment experience and well-being directly after media use. Next, we conducted a cross-sectional survey with two different populations; university students in the U.S. (*n* = 247) and adults in Germany (*n* = 289). Participants were asked to think of the last time they watched television for entertainment and report their entertainment experiences and well-being after media use. Finally, we aimed to replicate the previous results in a third survey (n = 291) using a different measure of hedonic and eudaimonic entertainment experiences in order to test if psychologically rich entertainment has been considered as a sub-component of eudaimonic entertainment in earlier work.

In all three studies, we also measured participants’ well-being during the last four weeks. We were able to replicate the findings by Oishi et al. [9] regarding the tripartite structure of well-being. Regarding entertainment experiences, however, we find mixed results. We see that psychologically rich entertainment is prevalent in everyday media use and has a positive relationship with well-being after media use. However, psychologically rich entertainment seems to overlap with eudaimonic entertainment to some extent; depending on the measurement approach, we find more or less of this overlap. In the following section, we introduce and explicate psychological richness as a unique predictor of entertainment experiences, and implications for understanding entertainment as a contributor to psychological well-being. Finally, we advocate for the development of a measurement scale for entertainment experiences that can more clearly distinguish between hedonic, eudaimonic, and psychologically rich entertainment.

### Psychological richness as a dimension of well-being

In order to define psychologically rich entertainment experiences, we will first briefly summarize the extant research on psychological richness as a dimension of well-being. Oishi and Westgate [10] describe a psychologically rich life as characterized by interest, variety, novelty, and perspective change. Such a life is facilitated by curiosity, time, energy and spontaneity, and leads to wisdom as an outcome. They contrast this to a happy life (hedonic well-being), which is characterized by comfort, joy, and security. The facilitators of a happy life are money, time, relationships and a positive mindset, the outcome is personal satisfaction. A meaningful life (eudaimonic well-being), in turn, is characterized by significance, purpose, and coherence. It is facilitated by moral principles, consistency, relationships and religiosity; the outcome of this way of life is societal contribution [10]. Psychological richness is thus distinct from hedonia and eudaimonia not only in terms of its qualities, but also its antecedents and consequences.

Oishi et al. [7] found that participants from nine countries perceive their daily lives as considerably rich; with the exception of Japan and Singapore, where the average rating of psychological richness was above the scale’s midpoint. However, ratings for happiness and meaning were higher across the board. In most countries, happiness was rated highest, followed by meaningfulness. When forced to choose between these types of lives, on average 14% of the population would prefer to lead a psychologically rich life over a happy or meaningful one. This desire was highest in Germany (16.8%) and lowest in Singapore (6.7%). In all of the investigated countries, however, people prefer to lead a happy life, followed by a meaningful one [7]. Hence, psychological richness is perceived as less important than hedonia and eudaimonia, but for a substantial part of the population, it reflects a preferred way of life. Moreover, even when psychological richness is not the priority, it still contributes to individuals’ overall well-being.

### Psychological richness as an entertainment experience

Entertainment is commonly defined as a pleasurable experience that includes physiological, cognitive, and affective components [26]. This definition comprises a variety of experiences, of which some refer to psychological richness. Bosshart and Macconi [15] for example, described six dimensions of entertainment; psychological relaxation, change and diversion, stimulation, fun, atmosphere and joy. Two of these dimensions–change/diversion and stimulation–clearly reflect experiences of psychological richness. Entertainment media content enables individuals to see the world through someone else’s eyes and to make vicarious experiences in domains that go well beyond the horizon of one’s personal experiences. In this vein, entertainment has also been conceptualized as a form of play that leads to a temporary change in perceived reality [20]. As can be seen from this brief overview, entertainment researchers have always considered the change in perspective as a key element that contributes to entertainment, but this aspect has been neglected so far when it comes to the measurement of outcomes of entertainment media use [14,21].

A second, but related, aspect is that media entertainment does not just provide new or different perspectives, but a broad variety of (vicarious) experiences. Bosshart and Macconi [15] describe different types of pleasure that can result from entertainment; the pleasure of the senses, ego-emotions, personal wit and knowledge, and socio-emotions. The first two of these pleasures describe how entertainment contributes to psychological richness through variety; individuals are confronted with diverse sensory impressions and can experience a broad range of emotions–from sadness or fear to happiness, and from suspense to relaxation [see also 26]. The experience of very different emotional states within a short time further contributes to what Besser and Oishi [7] describe as the “interest” component of psychological richness, which they see as a contrast to the potential boredom of emotional stability. Entertainment research has acknowledged that entertainment experiences consist of a great variety of emotions; however, the primary notion so far has been that these experiences are sought in order to reach a positively valenced state in the end (i.e., enjoyment [26]. While we do not want to invalidate this perspective, we propose that sometimes, the experience of diverse and unpleasant emotional states is not a means, but an end.

The third type of pleasure described by Bosshart and Macconi [15], the pleasure of personal wit and knowledge, is closely related to what Oishi and Westgate [10] describe as the outcome of a psychologically rich life: wisdom. By engaging with a variety of experiences, media users inevitably accumulate knowledge. This is a process that can be experienced as pleasurable itself, especially when it is relatively effortless [27]. Psychological richness can thus also be fostered by the possibility to learn something about a variety of things through entertainment media. Early studies on gratifications include some forms of gratification which could map on to the learning component of richness. For example, Herzog [28] listed educational appeal as a gratification of radio shows. Mendelsohn [29] suggested counteracting boredom and providing useful news and information as gratifications of radio use. Similarly, Greenberg [30] as well as Rubin [31,32] suggested learning about things or information was the key gratification sought from television.

In his seminal work on uses and gratifications from movies and television, Rubin [32] describes two types of television viewers; habitual viewers, who are driven to pass time and seek positive affect and diversion, and informational (later, instrumental) viewers. As Rubin [32] states: “The informational viewers are obviously not trying to escape from an information environment, but rather, are using television—and specific genres of informational programming—in order to learn about people, places and events and to instrumentally use this information in interpersonal interaction (a social interaction item that loaded highly on the information factor).” [32].

Although Rubin was specifically describing information-based viewers in this quote, the types of shows this viewing motive was associated with included talk-show, news, and game show programming, i.e., what a researcher today may yet consider entertainment television. In other words, Rubin demonstrated that the instrumental viewing motivation is distinct from escapist or pure hedonic motivations, but still in the context of leisure time television viewing. Thus, psychological richness has implicitly been considered as part of the entertainment experience since the early days of entertainment research. However, research on instrumental viewers, and the utility of richness, in an entertainment domain has been underemphasized in current models of entertainment.

The dichotomous perspective on entertainment, that was prevalent in the last decade, may have led scholars to define eudaimonic entertainment very broadly, subsuming different non-hedonic experiences under the umbrella of eudaimonia. For example, Bartsch [33] finds that a gratification of entertainment media is a contemplative emotional experience, which she links to the role of entertainment media in stimulating rewarding cognitive experiences. She states that this aspect is akin to appreciation or eudaimonic entertainment. We argue, however, that it is useful to distinguish between meaningful and psychologically rich entertainment experiences. Besser and Oishi [7] define psychological richness as “a life full of experiences which generate a state of mental engagement and arousal” (p. 1055). They note that such a life is characterized by complexity and the experience of a variety of interesting things and deep emotions. The focus of psychological richness–and hence psychologically rich entertainment–lies thus in the experience itself. Eudaimonic entertainment, on the other hand, is generated by the reflection of media users about such experiences. It requires a cognitive response to complex or challenging emotional states elicited by media content [22,25] and has thus also been described as resonance rather than an immediate entertainment experience [34,35]. As we will outline in the next paragraphs, psychological richness as a dimension of entertainment can help us to understand entertainment phenomena that are only partly explained by current two-factor perspectives on entertainment.

First, psychological richness as an entertainment experience accounts for the fact that entertainment is not always pleasurable, i.e., when watching a sad film or horror movie. Theories of hedonic entertainment postulate that negative emotions experienced during media use lead to arousal, which then may be attributed to enjoyment when the narration reaches a favorable outcome [36,37]. However, some genres, for example, sad films, never reach such an outcome. Theories of eudaimonic entertainment postulate that the experience of negative emotions during media use may transform into a positively valenced entertainment experience through meta appraisals, e.g., appreciation [26,38,39], or self-reflection [40]. This means that the negative emotions experienced during media use result in a positively valenced state because viewers appreciate the sad ending as a meaningful experience, or because they can reflect on their own experience. Psychological richness offers a complementary perspective; the experience of negative emotions during media use may be entertaining because these emotions are sought as a contrast to the emotional experiences of one’s everyday life, or as a novelty [10]. This perspective is especially fruitful to explain why people are entertained by pulp or camp horror movies, which are associated with intense negative emotions, but do not usually convey a deeper meaning or self-reflection.

Second, psychological richness may explain why people are entertained by documentaries and reality shows. The use of reality TV shows is mainly associated with hedonic entertainment; they foster the feeling of self-importance through perceptions of superiority, or by demonstrating that ordinary people can become celebrities [41]. While this explanation is well suited for shows such as *American Idol* or *The Bachelor*, it seems less fitting for shows such as *The Grand Tour* or *Queer Eye*, which are less likely to foster up- or downward comparisons. Rather, these shows allow the viewers to dive into a specific area of interest that may be very different from what they experience in their daily lives [42]. Rubin [32] noted that habitual viewers seek to escape reality for diversion, while informational viewers are drawn to real-life content such talk-shows, news and game show programming because of the value they see in learning about [other’s) reality. We argue that instrumental viewing is not solely linked to gaining knowledge about the world as an end goal, but can also come with an immediate gratification of satisfying one’s curiosity, which we consider to be an aspect of psychologically rich entertainment experiences.

Watching documentaries, on the other hand, has mostly been associated with eudaimonic entertainment. Many documentaries depict the beauty of nature or the interconnectedness of humanity and may thus be particularly moving or inspirational [43]. Yet, not all media users watch documentaries for self-transcendence or the search of a greater cause [18]; some just enjoy discovering a world outside of their personal experience, such as a different landscape, culture, or societal group. Or, they desire to expand their mind and knowledge on a given topic without necessarily finding greater meaning. Psychological richness may thus explain the use of real-world media content, i.e., reality TV and documentaries, better than hedonic and eudaimonic motivations.

Moreover, the concept of boundary expansion through media, under the model temporarily expanding the boundaries of the self (TEBOTS; [23,24]), could conceivably be construed as one way to achieve psychological richness. TEBOTS states that people desire experiences and perspectives that are outside their daily norm and the constraints of their own backgrounds, circumstances, and personalities, and media exposure allows them to briefly shed the bounds of the self and experience new things vicariously through characters. This is not necessarily a meaningful experience (e.g., the content is pure hedonic comedy), or an enjoyable one (e.g., the content is a tragedy in which all the main characters die at the end), but it *would* meet the requirement for psychologically rich entertainment.

However, in current understandings of entertainment, we do not see a superordinate factor emerging among scholars to account for these components of the informational viewer, i.e., interest, learning, and vicarious experience. Nor did Rubin link informational viewing to subsequent well-being outcomes, as we now do with hedonic and eudaimonic gratifications. Some aspects of psychologically rich entertainment, such as vicarious experience, boundary expansion, and food for thought have sometimes been considered as a sub-dimension of eudaimonic entertainment; however, mixing these aspects might veil their unique contributions to well-being. Therefore, our research aim is to examine psychologically rich entertainment as a dimension of entertainment experiences as important as, and at the same level to, hedonic and eudaimonic experiences.

## Study 1

### Research questions

Study 1 was designed to examine the following research questions within a naturalistic viewing environment.

RQ1: How is an individual’s general level of psychological richness related to the experience of hedonic, eudaimonic and psychologically rich entertainment during media use?
RQ2: How does the experience of hedonic entertainment, eudaimonic entertainment, and psychologically rich entertainment affect psychological richness after media use?
RQ3: What type of media content fosters the experience of psychologically rich entertainment?

### Method

#### Participants

Participants were recruited from an undergraduate communication course at a large Midwestern U.S. university between September 20, 2021, and December 1, 2021, and provided written consent before participation. All procedures and measures were approved by the institutional review board (IRB) of Michigan State University. Please see S1 File for Inclusivity in Global Research Questionnaire. Participation in the study was for course credit; students who did not want to participate completed a different assignment. From 37 participants who signed up for the study, 28 completed at least one viewing session with the related questionnaires. 20 were female, and 8 male; their age ranged from 19 to 25 (*M* = 21.00, *SD* = 1.09); 23 identified as White or Caucasian, 3 as Black or African American, and 2 reported other ethnicities.

#### Design and procedure

We conducted an experience sampling study, in which participants watched Netflix at home on their laptops and then responded to survey items using a browser extension for Chrome [44]. This extension was employed to track participants’ Netflix use during ten days; the application also triggered questionnaires directly after each viewing session. Upon installation of the browser extension, an initial survey was launched; next to socio-demographic information, participants reported their general level of hedonic well-being, eudaimonic well-being, and psychological richness during the last four weeks. After each viewing session, which means each time participants closed Netflix in their browser, they were asked to report their experience of hedonic, eudaimonic and psychologically rich entertainment during media use, and their current state of hedonic well-being, eudaimonic well-being, and psychological richness referring to the present day. In total, the participants completed 78 viewing sessions (ranging from 1 to 9 sessions per person) with related questionnaires during the study period.

#### Measures

**General well-being** was measured in reference to the past four weeks.

**General hedonic well-being** was measured with the SPANE questionnaire [45]; it consists of six positive items (e.g., “good”, “pleasant”) and six negative items (e.g., “bad”, “unpleasant”), participants indicate how often they feel these states on a scale from 1 (never/very rarely) to 5 (always/very often). Positive and negative values are summed up separately and the negative feelings score is then subtracted from the positive feelings score to create an affect balance score.

**General eudaimonic well-being** was measured with the meaning in life questionnaire [46], of which we used the presence subscale consisting of five items (e.g., “My life has a clear sense of purpose”), rated on a scale from 1 (absolutely untrue) to 7 (absolutely true).

**General psychological richness** was measured with the psychologically rich life questionnaire [8]; it consists of 12 items (e.g., “my life has been emotionally rich”, “I have had a lot of novel experiences”, which are rated on a scale from 1 (absolutely untrue) to 7 (absolutely true).

**Levels of hedonic and eudaimonic entertainment** were measured after each viewing session with 8 items from Wirth et al. [38; S1 Table].

**Psychologically rich entertainment** was measured with 3 items (“I enjoy that I have learned something new from watching this show”, “Watching this show has satisfied my curiosity”, “Watching this show has enriched my day”) reflecting experiences of psychological richness as described by Oishi et al. [8]. The first item addresses the experience of novelty, which Oishi et al. [8] identify as a core characteristic of situations that create psychological richness (e.g., p. 259). The second item refers to curiosity, which Oishi et al. [8] identify as a key personal characteristic that fosters psychological richness (p.259), or more precisely to the related gratification through media use. The third item reflects the fact that “individuals who lead a psychologically rich life seek to enrich their lives […] via travel, literature, film, music, sports, and the arts” [8; S1 Table].

**Situational well-being** was measured after each viewing session in reference to the present day. **State of hedonic well-being, eudaimonic well-being, and psychological richness** were measured with 9 items selected from a 15-item scale used by Oishi et al. [7]. The items (e.g., “happy”, “meaningful”, “interesting”) were introduced with “my day has been…” and were rated on a scale from 1 (not at all) to 7 (very much).

Means, standard deviations, and Cronbach’s Alpha values for all measures are listed in S2 Table.

**Age** (in years) and **gender** (male, female, non-binary) are included as control variables in all analyses.

### Results

To answer the first two research questions, we conducted multi-level regressions using the R-packages lme4, lmerTest, and performance. Since most participants had more than one viewing session within the study period, sessions were nested within participants to account for the repeated measurements. Bivariate correlations are depicted in S5 Table, and multilevel correlations of the constructs with repeated measures in S6 Table.

RQ1 addresses the relation between participants’ general level of psychological richness and their experience of hedonic, eudaimonic, and psychologically rich entertainment when watching Netflix. The entertainment experiences were included as dependent variables in three separate models, while general levels of hedonic well-being, eudaimonic well-being and richness were simultaneously included as independent variables, see S5 Table. All model coefficients are reported in Table 1. Psychological richness was positively related to hedonic entertainment, eudaimonic entertainment, as well as psychologically rich entertainment. General hedonic and eudaimonic well-being, on the other hand, were not related to any type of entertainment experience. Thus, individuals who perceive their life as psychologically rich are more likely to experience entertainment in general.

**Table 1:** Multilevel regression RQ1: Effect of psychological richness on entertainment experiences, study 1.

	Hedonic entertainment	Eudaimonic entertainment	Psychologically rich entertainment
***Fixed effects***			
**Intercept**	2.77 (1.17)*	2.05 (1.48)	2.72 (1.46)
**Hedonic well-being**	-0.00 (0.04)	0.06 (0.05)	0.05 (0.05)
**Eudaimonic well-being**	-0.22 (0.19)	-0.39 (0.23)	-0.42 (0.23)
**Psychological richness**	0.70 (0.22)**	0.72 (0.27)*	0.68 (0.27)*
***Random effects***			
**Intercept (SD)**	0.50 (0.70)	0.96 (0.98)	0.81 (0.90)
**Residual (SD)**	0.61 (0.78)	0.48 (0.70)	0.81 (0.90)
**ICC**	0.45	0.66	0.50
**Marginal R** ^**2**^	0.23	0.25	0.20

Note

* p < .05

** p < .01; fixed effects coefficients are estimates (standard errors), random effects coefficients are variances (standard deviations). Marginal R^2^ considers fixed effects only.

RQ2 addresses the effect of psychologically rich entertainment experiences on the state of hedonic well-being, eudaimonic well-being, and psychological richness after media use. We estimated separate models for each well-being state, including all three entertainment experiences as independent variables. A multi-level correlation analysis showed a significant positive relationship of all entertainment experiences, S6 Table; nevertheless, all three constructs were simultaneously included as independent variables. All model coefficients are reported in Table 2. The experience of psychologically rich entertainment is positively related to the state of psychological richness after the viewing session. The experience of eudaimonic entertainment was not related to well-being after media use, while the experience of hedonic entertainment was positively related to hedonic well-being. Thus, overall psychologically rich entertainment has a positive relationship not only with the experience of entertainment (see RQ1), but also with an individual’s well-being (RQ2).

**Table 2:** Multilevel regression RQ2: Effect of psychologically rich entertainment on well-being after media use, study 1.

	Hedonic well-being after media use	Eudaimonic well-being after media use	Psychologically rich well-being after media use
***Fixed effects***			
**Intercept**	2.93 (0.72)***	3.45 (0.70)***	2.20 (0.75)**
**Hedonic entertainment**	0.321 (0.14)*	-0.06 (0.13)	-0.18 (0.14)
**Eudaimonic entertainment**	-0.09 (0.16)	0.02 (0.16)	0.25 (0.17)
**Psychologically rich entertainment**	0.14 (0.15)	0.25 (0.14)	0.38 (0.16)*
***Random effects***			
**Intercept (SD)**	1.26 (1.12)	1.24 (1.11)	1.31 (1.14)
**Residual (SD)**	0.52 (0.72)	0.47 (0.68)	0.57 (0.75)
**ICC**	0.70	0.74	0.76
**Marginal R** ^**2**^	0.09	0.06	0.20

Note

* p < .05

** p < .01

*** < .001; fixed effects coefficients are estimates (standard errors), random effects coefficients are variances (standard deviations). Marginal R^2^ considers fixed effects only.

RQ3 addresses the type of media content that elicits psychologically rich entertainment. We created a table of all shows watched during the study period, and ranked them based on mean values of hedonic, eudaimonic, and psychologically rich entertainment reported after seeing the show (see OSF for the detailed results; https://bit.ly/3RtmAj3). Britney vs. Spears, Hip-Hop Evolution, and The Last Dance had the highest richness ratings; these are all documentaries. The lowest richness values were A Cinderella Story, Arrested Development, and Marvel’s Daredevil, which are fictional comedy or fantasy shows. Thus, we tentatively conclude that shows about real-life events or people, or educational content, may be linked to psychologically rich entertainment. It is important to note, however, that the values for psychologically rich entertainment are usually lower than those for hedonic or eudaimonic entertainment. This mirrors the findings of Oishi et al. [9], which show that the life of most individuals is happier and more meaningful than psychologically rich, but that psychological richness is nevertheless a substantial dimension of well-being.

### Discussion

In a naturalistic viewing environment, we found that psychological richness broadly related to experiencing entertainment—and did so more than hedonic or eudaimonic well being. In other words, viewers who experience psychological richness in their everyday life are likely to also seek out varied entertainment experiences of all types. Psychologically rich entertainment experiences are likely to lead to greater well-being in terms of richness post-viewing. Finally, in line with Rubin [32] we find that realistic programming is associated with psychological richness experiences—that is, shows that help us learn about the world or other people are experienced as uniquely enriching to everyday well-being.

## Study 2

### Study rationale and aims

Although we were able to provide some insight regarding the role of richness in entertainment consumption and effects, our method to capture naturalistic entertainment media use limited our sample size to a small set of participants, who were all from the same course at the same university in the U.S. In particular, the results related to the content watched should not be generalized beyond this specific population. Therefore, we wanted to replicate the initial findings from study 1 in a larger, more representative sample. Further, a larger sample also allows us to replicate the findings of Oishi and Westgate [10] with regard to the tripartite model of well-being, and to test if this structure is also suitable to conceptualize entertainment experiences. The research questions are the same as in study 1.

### Method

#### Design

We conducted two cross-sectional surveys, in which we asked participants to think about the last time they watched TV for entertainment. The survey was conducted with two different populations, 1) University students in the US, and 2) the general population in Germany. For each sample, we aimed for 250 participants based on an a-priori power analysis using G*Power. Based on the smallest effect obtained in study 1, we assumed a minimal effect size of R^2^ = 0.05 and selected an alpha error probability of 0.05 and 95% power, resulting in a required sample size of 249, which we rounded for convenience.

#### Participants

Participants in the US (*n* = 247) were recruited from the online participant pool at the same University as in study one between March 21, 2022, and April 20, 2022. Their age ranged from 18 to 63 (*M* = 19.96, *SD* = 3.20). Sixty-nine participants identified as male, 177 as female, and one person preferred their own terminology. Additionally, 177 were White or Caucasian, 21 Asian or Pacific Islanders, and 17 Black or African Americans. The remaining 32 participants reported other or multiple ethnicities. All participants provided written consent, and all procedures and measures were approved by the institutional research board of Michigan State University.

Participants in Germany (*n* = 289) were recruited from the online access panel of the market research company Bilendi from March 22–30, 2022. Participants received a small incentive (~1.5 €) for their participation in the study. Soft quotas were employed for age, gender and education in order to obtain a diverse sample resembling the general population. The age of participants ranged from 18 to 84 (*M* = 48.99, *SD* = 15.96), 135 identified as male and 154 as female. Forty-three participants had a University degree, 37 had a college qualification, 143 reported having a high school degree, 64 had completed mandatory school and 2 had no formal education. All participants provided written consent, and all procedures and measures were approved by the institutional research board of the University of Fribourg.

#### Procedure

In the survey, participants were first asked to report their general well-being during the last four weeks. Then, they were instructed to think about the last time they watched TV for entertainment (as opposed to information) and name the show(s) they had seen. All following questions then referred to this specific viewing session. Participants reported hedonic, eudaimonic and psychologically rich entertainment experiences while watching the show(s) and their well-being after media use using the same measures in Study 1. All constructs were measured as described in study 1. Means, standard deviations, and Cronbach’s Alpha values are listed in the S3 Table.

### Results

Bivariate correlations of all measured constructs are depicted in S7 Table. As a first step, we conducted confirmatory factors analysis (CFA) to inspect the tripartite models of well-being and entertainment S1 and S2 Figs. With regard to well-being, our data replicates the findings of Oishi and Westgate [10]; a model with three distinct factors for hedonic well-being, eudaimonic well-being, and psychological richness has a better fit to the data than a one-factor model or models with two factors (S9 Table). We report the fit indices of models with pooled data from our two samples, as a multi-group CFA resulted in a decreased fit. Overall, the tripartite model of well-being has a good fit to the data (*Χ*^*2*^ *=* 177.80, *df* = 24, *p* = .00, CFI = 0.96, SRMR = 0.05, RMSEA = 0.11), and thus a successful replication of the results of [10; S1 and S2 Figs].

We followed the same approach for entertainment. We compared the fit of a tripartite model consisting of hedonic entertainment, eudaimonic entertainment, and psychologically rich entertainment to a one-factor model comprising all entertainment dimensions and to a series of two-factor models. Again, the models have a better fit when applied to the pooled instead of the grouped data. The tripartite model has a satisfactory fit (*Χ*^*2*^ *=* 264.93, *df* = 41, *p* = .00 CFI = 0.94, SRMR = 0.06, RMSEA = 0.10) and performs better than the alternative models, see S10 Table. In this case, the traditional two-factor model, consisting of only hedonic and eudaimonic entertainment, does not have a better fit. Thus, our data suggest that psychological richness is a distinct component of entertainment experiences. Nevertheless, it must be noted that psychologically rich entertainment is strongly correlated to eudaimonic entertainment (*r* = .69, *p* < .01) and to hedonic entertainment (*r* = .56, *p* < .01), while hedonic and eudaimonic entertainment have a medium sized, significant correlation (*r* = .42, *p* < .01; S7 Table).

RQ1 addresses the relation between participants’ general level of psychological richness (trait) and their experience of hedonic, eudaimonic, and psychologically rich entertainment during media use. We estimated three regression models; each entertainment dimension was the dependent variable in one model, and all three well-being dimensions were simultaneously added as predictors. Despite the correlations of the variables (*r* = 0.33–0.52, S7 Table), there was no sign of multicollinearity in the models (VIF < 1.39). All model coefficients are reported in Table 3. Psychological richness had a positive relationship with hedonic entertainment, while age had a negative relationship. Hedonic well-being was also positively related to hedonic entertainment, but only marginally significant. Similarly, psychological richness had a positive relationship with eudaimonic entertainment, while eudaimonic well-being had a positive but only marginally significant relationship. Lastly, psychological richness was also positively related to psychologically rich entertainment, while age was negatively related. Overall, the analyses show that individuals who perceive their life as psychologically rich are more likely to experience entertainment during media use. Hedonic and eudaimonic well-being, on the other hand, had only marginally significant relations and only on the corresponding entertainment dimension. Although the causality of these relationships cannot be assessed with the data at hand, we note that once again richness has a unique relationship with entertainment.

**Table 3:** Regression models RQ1: Effect of general-well being on entertainment experiences, study 2.

	Hedonic entertainment	Eudaimonic entertainment	Psychologically rich entertainment
**Intercept**	4.95 (0.31)***	2.56 (0.38)***	4.00 (0.41)***
**Hedonic well-being**	0.01 (0.01)^t^	0.00 (0.01)	0.01 (0.01)
**Eudaimonic well-being**	0.01 (0.05)	0.10 (0.06)^t^	0.03 (0.06)
**Psychological richness**	0.19 (0.05)***	0.21 (0.06)***	0.16 (0.07)*
**Age**	-0.01 (0.00)***	0.00 (0.00)	-0.01 (0.00)*
**Gender**	0.16 (0.09)^t^	0.12 (0.11)	0.06 (0.12)
**N**	536	536	536
**R** ^**2**^	0.100	0.058	0.032
**R**^**2**^ **Adj.**	0.092	0.049	0.023

*Note*. Coefficients are unstandardized, values in brackets are the standard errors.

^t^ *p* < .10

* *p* < .05

** *p* < .01

*** *p* < .001

RQ2 addresses the relationship of psychologically rich entertainment experiences with the state of hedonic well-being, eudaimonic well-being, and psychological richness after media use. We estimated separate regression models for each well-being state, including all three entertainment experiences as independent variables. Again, despite the correlations of the entertainment dimensions, the models show no sign of severe multicollinearity (VIF < 2.28). All model coefficients are reported in Table 4. Hedonic well-being after media use was positively related to the experience of hedonic entertainment and eudaimonic entertainment. Eudaimonic well-being after media use was positively related to eudaimonic entertainment as well as psychologically rich entertainment. Psychological richness was positively related to the experience of psychologically rich entertainment and eudaimonic entertainment. Overall, we can conclude that entertainment experiences have a positive relationship with well-being after media use. The experience of psychologically rich entertainment, in particular, is positively related to psychological richness and eudaimonic well-being.

**Table 4:** Regression models RQ2: Effect of entertainment experiences on well-being after media use, study 2.

	Hedonic well-being after media use	Eudaimonic well-being after media use	Psychologically richness after media use
**Intercept**	2.05 (0.39)***	1.87 (0.37)***	1.81 (0.40)***
**Hedonic entertainment**	0.28 (0.06)***	0.06 (0.06)	0.06 (0.06)
**Eudaimonic entertainment**	0.19 (0.06)**	0.23 (0.06)***	0.26 (0.06)***
**Psychologically rich entertainment**	0.06 (0.06)	0.21 (0.06)***	0.19 (0.06)**
**Age**	-0.00 (0.00)	-0.00 (0.00)	0.00 (0.00)
**Gender**	0.04 (0.11)	0.02 (0.11)	-0.09 (0.12)
**N**	536	536	536
**R** ^**2**^	0.158	0.191	0.176
**R**^**2**^ **Adj.**	0.150	0.183	0.168

*Note*. Coefficients are unstandardized, values in brackets are the standard errors

^t^ *p* < .10

* *p* < .05

** *p* < .01

*** *p* < .001

RQ3 addresses the type of media content that elicits psychologically rich entertainment. We created a table of all shows watched during the study period, and calculated the mean values of reported hedonic entertainment, eudaimonic entertainment, and psychological richness across participants who had seen the same show. Shows that were seen by only one person were deleted from the list. The full table (and separated tables for the two samples) can be found on OSF (https://bit.ly/3RtmAj3). The highest values for richness were experienced while watching *Breaking Bad* (*M* = 6.44, *SD =* 0.96, *n* = 3), *Mord mit Aussicht* (a humorous crime show from Germany, *M* = 6.17, *SD =* 1.18, *n* = 2), and *Grey’s Anatomy* (*M* = 6.13, *SD* = 0.80, *n* = 8). However, all of these shows also scored quite high on hedonic entertainment as well as richness. Shows scoring highest on the richness dimension compared to the other two dimensions were *NCAA March Madness* (Basketball, *M* = 5.33, *SD* = 1.56, *n* = 4), *Terra X* (documentary, *M* = 5.33, *SD =* 0.47, *n* = 2), *Law and Order SVU* (*M* = 4.78, *SD* = 0.38, *n* = 3), and the news (unspecified news shows, (*M* = 4.5, *SD =* 1.00, *n* = 4). Thus, a wide range of TV shows do contribute to the experience of psychologically rich entertainment, and shows about real events seem to foster this dimension of entertainment in particular.

### Discussion

In study 2, we were able to replicate the findings from study 1 with regard to RQ1 and RQ2; individuals with high trait psychological richness are more likely to experience entertainment during media use, and the experience of psychologically rich entertainment contributes to well-being after media use. With regard to RQ3, the data from both samples suggest that informational content such as documentaries elicits more psychologically rich entertainment than hedonic or eudaimonic entertainment, but that psychologically rich entertainment can be experienced with a variety of TV shows. Our study is however only a first approach to this topic; the results must be interpreted with caution as they are based on a small number of participants who watched the same show. Future research is needed to better understand how psychological richness contributes to the selection of media content and to its effects.

Our data support the findings of Oishi et al. [9] who proposed a tripartite model of well-being, which includes hedonia, eudaimonia, and psychological richness as distinct dimensions. We show that this tripartite structure also applies to entertainment experiences. This is in line with early work of entertainment researchers, such as Bosshart and Macconi [15], who have referred to qualities of psychological richness as core features of entertainment experiences. It also extends early understandings of media selection motivations such as uses and gratifications [11], as well as validates primary components of Rubin’s [32] model of ritual versus instrumental viewers. Thus, we propose considering psychological richness as an important and distinct dimension of entertainment.

As a limitation, it has to be noted that this survey has a limited potential to capture experiences during media use, as it was not conducted during or directly after a viewing session. While the combination of studies 1 and 2 is well-suited to balance some of the limitations associated with each method, the cross-sectional nature of the data does not allow causal interpretations of the relations between entertainment experiences and well-being after media use in both cases. Thus, experimental research is needed to corroborate our findings.

Additionally, we found strong correlations between the entertainment dimensions; psychologically rich entertainment and eudaimonic entertainment were both associated with eudaimonic well-being and richness after media use. This indicates that the two concepts may not be sufficiently distinct when it comes to entertainment–either conceptually or in our approach to measure them.

## Study 3

### Study rationale

Despite the strong correlation of eudaimonic and psychologically rich entertainment, study 2 suggests a tripartite factor structure for entertainment experiences. However, this finding may be tied to the scale and items we chose to measure entertainment (i.e., a selection of items from Wirth et al. [21]). Another popular scale to measure eudaimonic entertainment responses is from Oliver and Bartsch [22]. This scale includes the dimensions of *fun*, which corresponds to hedonic entertainment, and *moving/thought provoking*, which corresponds to eudaimonic entertainment. One could argue that this conceptualization of eudaimonic entertainment is broader than the one by Wirth et al. [21], and that *thought provoking* refers to aspects of entertainment that we consider to be located within the concept of psychological richness.

### Research aims

The aim of study 3 is to replicate the findings from study 2 using a different measurement for hedonic and eudaimonic entertainment. Based on our theoretical expectations and the findings from studies 1 and 2, we formulated the following hypotheses:

H1: Trait-like psychological richness is positively related to the experience of a) hedonic, b) eudaimonic, and c) psychologically rich entertainment during media use.
H2: The experience of psychologically rich entertainment is positively related to the state of psychological richness after media use.
H3: A three-factor model of entertainment experiences (hedonic, eudaimonic, psychologically rich) has a better fit to the data than a one- or two-factor model.

### Method

#### Design

Study 3 was an observational survey study. The design, measures, and hypotheses were preregistered on OSF (https://bit.ly/3Hk8i0f). Study 3 was designed as a replication of study 2, therefore we opted to formulate hypotheses instead of the research questions that we used for studies 1 and 2.

#### Participants

Based on the same power calculation used in study 2, we aimed for a final sample of at least 250 participants, which we recruited from the same online participant pool as in studies 1 and 2. Ethics approval was obtained via the institutional review board at Michigan State University and all participants gave written consent before responding to any questions. In total, 359 participants completed the survey, we excluded those who responded less than 90% of the survey (n = 3), who reported being distracted, not remembering the last time they watched TV, or reported 0 hours and 0 minutes of entertainment consumption on an average day (n = 5), who failed the attention check (n = 43) and who reported more than one TV show for the open ended question (n = 17). The final sample consisted of 291 participants. Their age ranged from 18 to 38 (*M* = 19.87, *SD* = 1.92). 117 participants identified as male, 171 as female, and three persons as nonbinary. Additionally, 221 were White or Caucasian, 20 Black or African Americans, and 17 Asian or Pacific Islanders. The remaining 33 participants reported other or multiple ethnicities.

#### Procedure

The survey was identical to the one reported in study 2, except that we additionally included the items of Oliver and Bartsch [22] to measure entertainment. Means, standard deviations, and Cronbach’s Alpha values are listed in S4 Table.

### Results

Bivariate correlations of all measured constructs are depicted in S8 Table. To test H1, we estimated the same regression models as in study 2; we included all three dimensions of well-being simultaneously as predictors of entertainment experiences despite their substantial correlations (*r* = 0.29–0.49, S8 Table) as there was no sign of multicollinearity (VIF < 1.56). All model coefficients are reported in Table 5. Psychological richness had a positive relationship with hedonic entertainment and psychologically rich entertainment, but unlike in studies 1 and 2 not with eudaimonic entertainment. Surprisingly, eudaimonic entertainment was negatively related to hedonic well-being. Thus, H1a and H1c are supported, but H1b is not. While the relationship between psychological richness and the experience of entertainment during media use is less exclusive with this measurement approach, we can still conclude that a person’s general level of psychological richness is positively associated with the experience of entertainment.

**Table 5:** Regression models H1a-c: Effect of general-well being on entertainment experiences, study 3.

	Hedonic entertainment	Eudaimonic entertainment	Psychologically rich entertainment
**Intercept**	5.48 (0.56)***	4.16 (0.98)***	3.54 (1.01)***
**Hedonic well-being**	0.00 (0.01)	-0.04 (0.01)**	-0.01 (0.01)
**Eudaimonic well-being**	0.04 (0.05)	0.11 (0.08)	0.12 (0.08)
**Psychological richness**	0.14 (0.05)**	0.13 (0.09)	0.20 (0.09)*
**Age**	-0.01 (0.02)	-0.02 (0.04)	-0.01 (0.04)
**Gender**	-0.12 (0.09)	-0.06 (0.16)	-0.15 (0.17)
**N**	290	290	290
**R** ^**2**^	0.053	0.034	0.043
**R**^**2**^ **Adj.**	0.037	0.017	0.027

*Note*. Coefficients are unstandardized, values in brackets are the standard errors.

^t^ *p* < .10

* *p* < .05

** *p* < .01

*** *p* < .001

To test the relationship of entertainment experiences on well-being after media use, we also included all three dimensions of entertainment simultaneously, as the models show no sign of severe multicollinearity (VIF < 1.99). All model coefficients are reported in Table 6. Hedonic well-being after media use was positively related to all three dimensions of entertainment. Eudaimonic well-being after media use was predicted by psychologically rich entertainment, while psychological richness after media use was not related to any entertainment dimension. H2 is thus not supported by the data; the effects of entertainment on well-being after media use are less consistent to theoretical expectations when entertainment is measured with the items of Oliver and Bartsch [22], but psychological richness during media use is still significantly related to well-being after media use.

**Table 6:** Regression models H2: Effect of entertainment experiences on well-being after media use, study 3.

	Hedonic well-being after media use	Eudaimonic well-being after media use	Psychologically richness after media use
**Intercept**	3.86 (0.83)***	4.77 (0.97)***	3.66 (1.01)**
**Hedonic entertainment**	0.25 (0.07)**	-0.04 (0.08)	-0.04 (0.08)
**Eudaimonic entertainment**	0.01 (0.07)**	0.07 (0.08)	0.14 (0.09)
**Psychologically rich entertainment**	0.07 (0.06)**	0.19 (0.08)**	0.15 (0.08)^t^
**Age**	-0.03 (0.04)	-0.07 (0.04)^t^	-0.04 (0.04)
**Gender**	0.03 (0.13)	-0.05 (0.15)	0.00 (0.16)
**N**	290	290	290
**R** ^**2**^	0.079	0.069	0.065
**R**^**2**^ **Adj.**	0.063	0.053	0.049

*Note*. Coefficients are unstandardized, values in brackets are the standard errors.

^t^ *p* < .10

* *p* < .05

** *p* < .01

*** *p* < .001

To test H3 we conducted confirmatory factors analysis (CFA) comparing the fit of a tripartite model to a one-factor model and to a series of two-factor models. The tripartite model has a good fit (*Χ*^*2*^ *=* 69.739, *df* = 24, *p* = .00, CFI = 0.97, SRMR = 0.04, RMSEA = 0.08) and performs better than the alternative models S11 Table, S3 Fig., H3 is thus supported. However, the two-factor model only containing the dimensions fun and moving has a better fit than all models including psychological richness. Hence, also when using a different measurement approach for hedonic and eudaimonic well-being, psychological richness proves to be a distinct component of entertainment experiences. However, the scale of Oliver and Bartsch performs better on its own than in combination with the richness items.

### Discussion

The aim of study 3 was to test if the tripartite structure of entertainment experiences and their relationship with well-being can be replicated when using a different operationalization of hedonic and eudaimonic entertainment. Overall, the data support that psychological richness constitutes a predictor of entertainment, is a distinct component of entertainment experiences, and affects well-being after media use. However, the relationships between the dimensions of well-being and entertainment are less consistent with theoretical expectations when using the items of Oliver & Bartsch [22] compared to those of Wirth et al. [21]. Unexpected effects were not only found in relation to psychological richness, but for all three dimensions. Again, we found a strong correlation of psychologically rich entertainment with eudaimonic entertainment (*r* = .68, *p* < .01), but a less strong correlation with hedonic entertainment (*r* = .39, *p* < .01). Overall, the findings of study 3 clearly indicate that the operationalization of entertainment experiences matters when studying relationships with well-being. Hence, we suggest paying particular attention to this aspect when further developing the measurement of psychologically rich entertainment experiences.

## General discussion

Based on recent developments in the conceptualization of psychological well-being, we examined the role of psychological richness in both selection of and response to entertainment media in both an experience sampling study and two surveys. We were able to replicate the tripartite structure of well-being as proposed by Oishi et al. [9], and find that such a structure is also suitable when it comes to entertainment experiences. However, psychologically rich entertainment is strongly correlated to eudaimonic entertainment, suggesting a certain overlap between these constructs. We argue below that this is less of a theoretical or conceptual problem, and instead one of operationalization.

Our results additionally show that media users do experience psychologically richness via entertainment, and that this experience has a positive relationship with well-being after media use. Further, we found that individuals who perceive their lives as psychologically rich are more likely to be entertained during media use. This latter finding might reflect that psychological richness requires a certain openness to experiences, which could foster the immersion into a narration and thus entertainment experiences. Hence, the investigation of trait-like psychological richness in the context of media entertainment constitutes a promising avenue for future research.

Our findings, in line with early entertainment work, underscore the question: Why has this type of viewing motivation not been considered as a distinct construct in recent entertainment research? We do often see learning or information appear in early stages of studies, but disappear or become subsumed in later work. For example, Bartsch and Viehoff [47] listed learning and social motivations for entertainment in their model for experiencing emotions from entertainment. In a follow-up study [33], thought-provoking does not emerge as a unique factor of emotional gratifications from media, but is integrated into the contemplative emotional experiences factor, which Bartsch links to appreciation (and thus eudaimonic gratifications). Nevertheless, she mentions the need to distinguish “between the gratification derived from specific feelings associated with the tear-jerker genre (i.e., sad and poignant feelings), and the cognitively stimulating function of negative and mixed emotions that include but are not limited to sadness and poignancy.” [33].

We argue that psychological richness as a dimension of entertainment experiences refers to this latter aspect of why individuals seek intense emotional experiences, also of negative valence, and why they are entertained by real life content (such as reality TV and documentaries). Indeed, in our study we find that the experience of psychologically rich entertainment is not limited to informational or reality-based content; a great variety of suspenseful, emotional, and even funny TV shows elicited high levels of psychologically rich entertainment.

We feel that perhaps the reason much work on entertainment motivations has not separated out informational or richness-based viewing of entertainment is due to the lack of clear explication of the term. Indeed, if we look at prior studies, we see the same idea termed information [11], instrumental [48], learning [32], vicarious experience [41], and so on. In this case, the tendency of communication researchers to lean on poorly explicated or overlapping concepts has perhaps led to conceptual obfuscation. We would suggest that richness, rather than adding to the confusion, may instead bring clarity to a set of interrelated gratifications from entertainment that have hitherto been under-emphasized in entertainment work.

It could also be that the ritual/instrumental dichotomy has been mired in the debate between active and passive viewing. Uses and gratifications assumes an active audience, who actively chooses what content and setting to view. On the other hand, entertainment experiences generally have been mapped onto the more passive viewing of habitual viewers—for example, the notions of couch potatoes, binge viewers, and other such terms being used to describe non-motivated, passive or escapist use of entertainment for diversion. See for example Warren [49] who suggests that media affordances must be better conceptualized in order to understand the dichotomy between ritual and instrumental viewers.

It could also be that there is an inherent bias in how entertainment is studied—that is, as a leisure time activity with no real value—that has led researchers to categorize learning from entertainment as a lesser motivation for consumption. In comparison to say, political communication scholars, who have extensively examined the role of information-as-entertainment [50–53], entertainment scholars seem to be less interested in entertainment-as-information. There is a wealth of literature examining edutainment, or the inclusion of educational content in entertainment [54,55], but the notion of simply watching because learning is pleasurable seems to have fallen by the wayside in entertainment media research.

However, our work demonstrates that psychologically rich entertainment is not only important for leisure viewing, but also for subsequent well-being. Much in the way that understanding eudaimonic motivations for entertainment consumption led to a large body of work on meaning from entertainment [17,18,56], we are hopeful that understanding the role of psychological richness may be equally fruitful in explaining and streamlining existing motivations for entertainment consumption. This can open up new perspectives on how entertainment media contribute to well-being. While it is beyond the scope of this paper to investigate the processes through which entertainment media affect well-being (e.g., learning, emotions, etc.), we demonstrate that there is a link between different dimensions of entertainment experiences and corresponding dimensions of well-being.

Our studies consistently show a high correlation of psychologically rich entertainment and eudaimonic entertainment, and subsequently positive relations with both types of well-being after media use. This may reflect that some aspects of psychologically rich entertainment have been considered by earlier work on eudaimonic entertainment. We argue that it is useful to distinguish between eudaimonic and psychologically rich aspects of entertainment and well-being. As we have discussed earlier in the manuscript, psychologically rich entertainment refers to an experience where emotions–also negative ones–provide an immediate entertainment gratification, whereas eudaimonic entertainment is the outcome of a reflection process in which negative emotions transform into positive meta-emotions. Our findings suggest that rather than just adding items to capture psychologically rich entertainment to existing scales that measure hedonic and eudaimonic entertainment, it would be advisable to develop a new scale that clearly differentiates between eudaimonic and psychologically rich entertainment, also from a theoretical perspective. Thereby, we would suggest paying more attention to the different sub-dimensions of psychologically rich entertainment experiences and seeking to differentiate between mentally and emotionally engaging experiences.

The high correlations between eudaimonic and psychologically rich entertainment might also reflect that the experience of psychologically rich entertainment can (but not necessarily has to) be transformed into subsequent eudaimonic entertainment, and thus contribute to both dimensions of well-being. Even if this is the case, however, that does not mean that they are not separate constructs that are worth studying on their own. Future research should therefore further investigate the relationship of entertainment experiences from a process perspective. Another fruitful avenue in this direction would be to consider the role of cognitive and affective challenge in relation to eudaimonic and psychologically rich entertainment, or by examining the unique contribution of psychological needs to diverse entertainment experiences.

Our research provides first insights regarding the media content that is associated with psychologically rich entertainment experiences. While study 1 tentatively supports our assumption that real-life content fosters psychologically entertainment experiences, this pattern was not found in study 2. Further, we assumed that content evoking intense, yet non-meaningful, negative emotions, such as horror movies, would also be associated with psychological richness. Due to the limited consumption of horror content among our participants, we are not able to draw any conclusions with regard to this claim. Future research is needed to investigate, likely in experimental settings, which types of media content particularly foster psychologically rich entertainment, and do so via behavioral measures. Experimental studies with prototypical content can also contribute to the development of a measurement that allows to differentiate between the three proposed dimensions of entertainment experiences more accurately, and elucidate the gratifications (cognitive and emotional) that are linked to psychologically rich entertainment.

### Conclusion

This research makes an important contribution to the entertainment literature, in that it demonstrates that psychological richness is prevalent in everyday media use and that this entertainment experience has a positive impact on well-being. The relationships of psychologically rich entertainment and well-being offer two promising avenues for future research: 1) The positive relationship between general psychological well-being and the experience of all three types of entertainment warrants closer attention. 2) The positive relationship between psychologically rich entertainment and well-being after media use needs further investigation, not only with regard to causality, but also with regard to potential moderators and mediators. Before such relationships can be investigated, it is however important to develop a scale to measure all three dimensions of entertainment as distinct constructs. Regardless, we argue that psychological richness deserves further scrutiny as an entertainment experience on the same level as hedonic and eudaimonic experiences.

## Supporting information

S1 Table Measurement of entertainment experiences in study 1, 2, and 3. (DOCX)

S2 Table Means, standard deviations, and Cronbach’s Alpha for study 1. (DOCX)

S3 Table Means, standard deviations, and Cronbach’s Alpha for study 2. (DOCX)

S4 Table Means, standard deviations, and Cronbach’s Alpha for study 3. (DOCX)

S5 Table Bivariate correlations, study 1. *Note*. * indicates *p* < .05. ** indicates *p* < .01.
(DOCX)

S6 Table Multilevel correlations of variables with repeated measures, study 1. *Note*.* indicates *p* < .05. ** indicates *p* < .01.
(DOCX)

S7 Table Bivariate correlations, study 2. *Note*. * *p* < .05, ** *p* < .01.
(DOCX)

S8 Table Bivariate correlations, study 3. *Note*. * *p* < .05, ** *p* < .01.
(DOCX)

S9 Table CFA model fit indices for well-being, study 2. Note. CFI = comparative fit index, SRMR = standardized root mean square residual, RMSEA = root mean square error of approximation.
(DOCX)

S10 Table CFA model fit indices for entertainment, study 2. *Note*. CFI = comparative fit index, SRMR = standardized root mean square residual, RMSEA = root mean square error of approximation.
(DOCX)

S11 Table CFA model fit indices for entertainment, study 3. *Note*. CFI = comparative fit index, SRMR = standardized root mean square residual, RMSEA = root mean square error of approximation.
(DOCX)

S1 Fig Factor loadings for the tripartite model of well-being (study 2). (DOCX)

S2 Fig Factor loadings for the tripartite model of entertainment (study 2). (DOCX)

S3 Fig Factor loadings for the tripartite model of entertainment (study 3). (DOCX)

S1 File Inclusivity in global research response file. (DOCX)
