# Supplementary material for: Beyond pleasurable and meaningful: Psychologically rich entertainment experiences
Source: PLoS One. 2025 Feb 6;20(2):e0315596. doi: 10.1371/journal.pone.0315596 (PMC11801586; doi:10.1371/journal.pone.0315596)
Supplement: S1 Table — (DOCX) [file pone.0315596.s001.docx]

**S1 Table.** **Measurement of Entertainment Experiences in Study 1, 2, and 3.**

| Study 1 & 2 | **Hedonic entertainment (Wirth et al. 2012)** |
| --- | --- |
|  | I felt well entertained watching the show. |
|  | Watching the show was fun. |
|  | It gave me pleasure to watch the show. |
|  | **Eudaimonic entertainment (Wirth et al. 2012)** |
|  | The show made me realize how content I can be with my own life. |
|  | Now that I have seen this show, I feel that I am in charge of my own life. |
|  | The emotions that I felt during the show challenged me in a positive way. |
|  | It felt good to be captivated by the events around the characters during the show. |
|  | I had the feeling that the show delivered central values of life in an authentic way. |
|  | **Psychologically rich entertainment** |
|  | I enjoy that I have learned something new from watching this show. |
|  | Watching this show has satisfied my curiosity. |
|  | Watching this show has enriched my day. |
| Study 3 | **Hedonic entertainment (fun) (Oliver & Bartsch 2010)** |
|  | It was fun for me to watch [name of the show]. |
|  | I had a good time watching [name of the show]. |
|  | [name of the show] was entertaining. |
|  | **Eudaimonic entertainment (moving) (Oliver & Bartsch 2010)** |
|  | I found [name of the show] to be very meaningful. |
|  | I was moved by [name of the show]. |
|  | [name of the show] was thought provoking. |
|  | **Psychologically rich entertainment** |
|  | I enjoy that I have learned something new from watching this show. |
|  | Watching this show has satisfied my curiosity. |
|  | Watching this show has enriched my day. |
