# Supplementary material for: Beyond pleasurable and meaningful: Psychologically rich entertainment experiences
Source: PLoS One. 2025 Feb 6;20(2):e0315596. doi: 10.1371/journal.pone.0315596 (PMC11801586; doi:10.1371/journal.pone.0315596)
Supplement: S2 Table — (DOCX) [file pone.0315596.s002.docx]

**S2 Table. Means, Standard Deviations, and Cronbach’s Alpha for Study 1.**

| Variable | *M* | *SD* | *Cronbach’s Alpha* |
| --- | --- | --- | --- |
| Hedonic well-being | 7.32 | 4.33 | 0.79 |
| Eudaimonic well-being | 4.74 | 1.01 | 0.80 |
| Psychological richness | 5.33 | 0.82 | 0.88 |
| Hedonic entertainment | 5.35 | 1.23 | 0.95 |
| Eudaimonic entertainment | 4.31 | 1.42 | 0.97 |
| Psychological richness | 4.62 | 1.41 | 0.96 |
| Hedonic well-being after media use | 4.79 | 1.18 | 0.97 |
| Eudaimonic well-being after media use | 4.31 | 1.20 | 0.98 |
| Psychological richness after media use | 3.98 | 1.63 | 0.98 |

*Note.* For entertainment and state measures of well-being, the calculation of Cronbach’s Alpha considers the multi-level structure of the data; the reported value is RkRn, which is suitable when measurements are nested in individuals and the time of measurement varies [(Revelle & Condon, 2019)](https://www.zotero.org/google-docs/?YF4lRA).
