# Supplementary material for: Beyond pleasurable and meaningful: Psychologically rich entertainment experiences
Source: PLoS One. 2025 Feb 6;20(2):e0315596. doi: 10.1371/journal.pone.0315596 (PMC11801586; doi:10.1371/journal.pone.0315596)
Supplement: S3 Table — (DOCX) [file pone.0315596.s003.docx]

**S3 Table. Means, Standard Deviations, and Cronbach’s Alpha for Study 2.**

| Variable | *M* | *SD* | *Cronbach’s Alpha* |
| --- | --- | --- | --- |
| Hedonic well-being | 6.34 | 7.16 | .89 |
| Eudaimonic well-being | 4.75 | 1.20 | .85 |
| Psychological richness | 5.17 | 1.03 | .92 |
| Hedonic well-being after media use | 4.86 | 1.37 | .92 |
| Eudaimonic well-being after media use | 4.20 | 1.35 | .83 |
| Psychological richness after media use | 4.16 | 1.41 | .84 |
| Hedonic entertainment | 5.89 | 1.10 | .93 |
| Eudaimonic entertainment | 4.43 | 1.31 | .86 |
| Psychologically rich entertainment | 4.85 | 1.41 | .84 |
