# Supplementary material for: Beyond pleasurable and meaningful: Psychologically rich entertainment experiences
Source: PLoS One. 2025 Feb 6;20(2):e0315596. doi: 10.1371/journal.pone.0315596 (PMC11801586; doi:10.1371/journal.pone.0315596)
Supplement: S4 Table — (DOCX) [file pone.0315596.s004.docx]

**S4 Table. Means, Standard Deviations, and Cronbach’s Alpha for Study 3.**

| Variable | *M* | *SD* | *Cronbach’s Alpha* |
| --- | --- | --- | --- |
| Hedonic well-being | 7.30 | 6.75 | .70 |
| Eudaimonic well-being | 4.74 | 1.24 | .87 |
| Psychological richness | 5.17 | 1.05 | .92 |
| Hedonic well-being after media use | 5.05 | 1.18 | .85 |
| Eudaimonic well-being after media use | 4.23 | 1.37 | .84 |
| Psychological richness after media use | 3.87 | 1.42 | .80 |
| Hedonic entertainment (fun) | 6.16 | 0.83 | .88 |
| Eudaimonic entertainment (moving) | 4.55 | 1.41 | .83 |
| Psychologically rich entertainment | 4.65 | 1.46 | .86 |
