# Supplementary material for: Beyond pleasurable and meaningful: Psychologically rich entertainment experiences
Source: PLoS One. 2025 Feb 6;20(2):e0315596. doi: 10.1371/journal.pone.0315596 (PMC11801586; doi:10.1371/journal.pone.0315596)
Supplement: S5 Table — Note. * indicates p < .05. ** indicates p < .01. (DOCX) [file pone.0315596.s005.docx]

**S5 Table. Bivariate correlations, study 1.** *Note.* * indicates *p* < .05. ** indicates *p* < .01.

| Variable | 1 | 2 | 3 | 4 | 5 | 6 | 7 | 8 |
| --- | --- | --- | --- | --- | --- | --- | --- | --- |
| 1. Hedonic well-being |  |  |  |  |  |  |  |  |
| 2. Eudaimonic well-being | .31 |  |  |  |  |  |  |  |
| 3. Psychological richness | .30 | .30 |  |  |  |  |  |  |
| 4. Hedonic entertainment | .03 | .03 | .52** |  |  |  |  |  |
| 5. Eudaimonic entertainment | .21 | -.14 | .37 | .66** |  |  |  |  |
| 6. Psychologically rich entertainment | .00 | -.15 | .33 | .64** | .82** |  |  |  |
| 7. Hedonic well-being after media use | .20 | -.11 | .15 | .21 | .20 | .35 |  |  |
| 8. Eudaimonic well-being after media use | .30 | -.28 | -.06 | -.03 | .37 | .39* | .61** |  |
| 9. Psychological richness  after media use | .23 | -.43* | -.12 | .08 | .48** | .49** | .46* | .82** |
