# Supplementary material for: Beyond pleasurable and meaningful: Psychologically rich entertainment experiences
Source: PLoS One. 2025 Feb 6;20(2):e0315596. doi: 10.1371/journal.pone.0315596 (PMC11801586; doi:10.1371/journal.pone.0315596)
Supplement: S6 Table — Note.* indicates p < .05. ** indicates p < .01. (DOCX) [file pone.0315596.s006.docx]

**S6 Table. Multilevel correlations of variables with repeated measures, Study 1.** *Note.* * indicates *p* < .05. ** indicates *p* < .01.

| Variable | 1 | 2 | 3 | 4 | 5 |
| --- | --- | --- | --- | --- | --- |
| 1. Hedonic entertainment |  |  |  |  |  |
| 2. Eudaimonic entertainment | .60** |  |  |  |  |
| 3. Psychologically rich entertainment | .61** | .83** |  |  |  |
| 4. Hedonic well-being after media use | .39** | .36** | .43** |  |  |
| 5. Eudaimonic well-being after media use | .27* | .44** | .46** | .83** |  |
| 6. Psychological richness after media use | .26* | .51** | .53** | .70** | .87** |
