# Supplementary material for: Beyond pleasurable and meaningful: Psychologically rich entertainment experiences
Source: PLoS One. 2025 Feb 6;20(2):e0315596. doi: 10.1371/journal.pone.0315596 (PMC11801586; doi:10.1371/journal.pone.0315596)
Supplement: S7 Table — Note. * p < .05, ** p < .01. (DOCX) [file pone.0315596.s007.docx]

**S7 Table. Bivariate Correlations, Study 2.** *Note.* * *p* < .05, ** *p* < .01.

| Variable | 1 | 2 | 3 | 4 | 5 | 6 | 7 | 8 |
| --- | --- | --- | --- | --- | --- | --- | --- | --- |
| 1. Hedonic well-being |  |  |  |  |  |  |  |  |
| 2. Eudaimonic well-being | .52** |  |  |  |  |  |  |  |
| 3. Psychological richness | .33** | .47** |  |  |  |  |  |  |
| 4. Hedonic well-being after media use | .50** | .35** | .30** |  |  |  |  |  |
| 5. Eudaimonic well-being after media use | .39** | .38** | .31** | .73** |  |  |  |  |
| 6. Psychological richness after media use | .25** | .27** | .29** | .54** | .76** |  |  |  |
| 7. Hedonic entertainment | .15** | .11* | .21** | .34** | .27** | .24** |  |  |
| 8. Eudaimonic entertainment | .12** | .19** | .22** | .32** | .39** | .39** | .42** |  |
| 9. Psychologically rich entertainment | .09* | .09* | .14** | .32** | .40** | .37** | .56** | .69** |
