# Supplementary material for: Beyond pleasurable and meaningful: Psychologically rich entertainment experiences
Source: PLoS One. 2025 Feb 6;20(2):e0315596. doi: 10.1371/journal.pone.0315596 (PMC11801586; doi:10.1371/journal.pone.0315596)
Supplement: S8 Table — Note. * p < .05, ** p < .01. (DOCX) [file pone.0315596.s008.docx]

**S8 Table. Bivariate Correlations, Study 3.** *Note.* * *p* < .05, ** *p* < .01

| Variable | 1 | 2 | 3 | 4 | 5 | 6 | 7 | 8 |
| --- | --- | --- | --- | --- | --- | --- | --- | --- |
| 1. Hedonic well-being |  |  |  |  |  |  |  |  |
| 2. Eudaimonic well-being | .46** |  |  |  |  |  |  |  |
| 3. Psychological richness | .29** | .49** |  |  |  |  |  |  |
| 4. Hedonic well-being after media use | .44** | .36** | .25** |  |  |  |  |  |
| 5. Eudaimonic well-being after media use | .30** | .37** | .27** | .63** |  |  |  |  |
| 6. Psychological richness after media use | .18** | .32** | .28** | .42** | .70** |  |  |  |
| 7. Hedonic entertainment (fun) | .10 | .15** | .21** | .23** | .11 | .11 |  |  |
| 8. Eudaimonic entertainment (moving) | -.10 | .06 | .09 | .01 | .17** | .23** | .29** |  |
| 9. Psychologically rich entertainment | .06 | .16** | .18** | .18** | .24** | .23** | .39** | .68** |
