# Supplementary material for: Beyond pleasurable and meaningful: Psychologically rich entertainment experiences
Source: PLoS One. 2025 Feb 6;20(2):e0315596. doi: 10.1371/journal.pone.0315596 (PMC11801586; doi:10.1371/journal.pone.0315596)
Supplement: S9 Table — Note. CFI = comparative fit index, SRMR = standardized root mean square residual, RMSEA = root mean square error of approximation. (DOCX) [file pone.0315596.s009.docx]

**S9 Table. CFA model fit indices for well-being, Study 2.** Note. CFI = comparative fit index, SRMR = standardized root mean square residual, RMSEA = root mean square error of approximation

| Model | CFI | SRMR | RMSEA | *Χ^2^* | *df* |
| --- | --- | --- | --- | --- | --- |
| One-factor model | 0.816 | 0.086 | 0.212 | 675.694 | 27 |
| Two-factor models |  |  |  |  |  |
| - F1 hedonic + richness, F2 eudaimonic | 0.823 | 0.095 | 0.211 | 649.297 | 26 |
| - F1 eudaimonic + richness, F2 hedonic | 0.931 | 0.060 | 0.132 | 269.884 | 26 |
| - F1 hedonic + eudaimonic, F2 richness | 0.874 | 0.082 | 0.179 | 471.879 | 26 |
| - F1 hedonic, F2 eudaimonic | 0.983 | 0.027 | 0.097 | 48.284 | 8 |
| Three-factor model | 0.956 | 0.050 | 0.109 | 177.800 | 24 |
