# Supplementary material for: Beyond pleasurable and meaningful: Psychologically rich entertainment experiences
Source: PLoS One. 2025 Feb 6;20(2):e0315596. doi: 10.1371/journal.pone.0315596 (PMC11801586; doi:10.1371/journal.pone.0315596)
Supplement: S10 Table — Note. CFI = comparative fit index, SRMR = standardized root mean square residual, RMSEA = root mean square error of approximation. (DOCX) [file pone.0315596.s010.docx]

**S10 Table. CFA model fit indices for entertainment, Study 2.** *Note.* CFI = comparative fit index, SRMR = standardized root mean square residual, RMSEA = root mean square error of approximation

| Model | CFI | SRMR | RMSEA | *Χ^2^* | *df* |
| --- | --- | --- | --- | --- | --- |
| One-factor model | 0.671 | 0.113 | 0.232 | 1315.790 | 44 |
| Two-factor models |  |  |  |  |  |
| - F1 hedonic + richness, F2 eudaimonic | 0.796 | 0.135 | 0.185 | 830.959 | 43 |
| - F1 eudaimonic + richness, F2 hedonic | 0.897 | 0.071 | 0.132 | 441.855 | 43 |
| - F1 hedonic + eudaimonic, F2 richness | 0.701 | 0.159 | 0.224 | 1198.582 | 43 |
| - F1 hedonic, F2 eudaimonic | 0.946 | 0.062 | 0.118 | 161.635 | 19 |
| Three-factor model | 0.942 | 0.056 | 0.101 | 264.934 | 41 |
