# Supplementary material for: Beyond pleasurable and meaningful: Psychologically rich entertainment experiences
Source: PLoS One. 2025 Feb 6;20(2):e0315596. doi: 10.1371/journal.pone.0315596 (PMC11801586; doi:10.1371/journal.pone.0315596)
Supplement: S11 Table — Note. CFI = comparative fit index, SRMR = standardized root mean square residual, RMSEA = root mean square error of approximation. (DOCX) [file pone.0315596.s011.docx]

**S11 Table. CFA model fit indices for entertainment, Study 3.** *Note.* CFI = comparative fit index, SRMR = standardized root mean square residual, RMSEA = root mean square error of approximation

| Model | CFI | SRMR | RMSEA | *Χ^2^* | *df* |
| --- | --- | --- | --- | --- | --- |
| One-factor model | 0.655 | 0.140 | 0.256 | 543.751 | 27 |
| Two-factor models |  |  |  |  |  |
| - F1 hedonic (fun) + richness, F2 eudaimonic (moving) | 0.675 | 0.169 | 0.254 | 512.492 | 26 |
| - F1 eudaimonic (moving) + richness, F2 hedonic | 0.919 | 0.058 | 0.127 | 147.558 | 26 |
| - F1 hedonic (fun) + eudaimonic, F2 richness | 0.686 | 0.191 | 0.249 | 496.219 | 26 |
| - F1 hedonic, F2 eudaimonic | 0.999 | 0.020 | 0.020 | 8.936 | 8 |
| Three-factor model | 0.969 | 0.042 | 0.081 | 69.739 | 24 |
