# Supplementary figures and images for: Beyond pleasurable and meaningful: Psychologically rich entertainment experiences
Source: PLoS One. 2025 Feb 6;20(2):e0315596. doi: 10.1371/journal.pone.0315596 (PMC11801586; doi:10.1371/journal.pone.0315596)

**S1 Fig. Factor loadings for the tripartite model of well-being (study 2)**


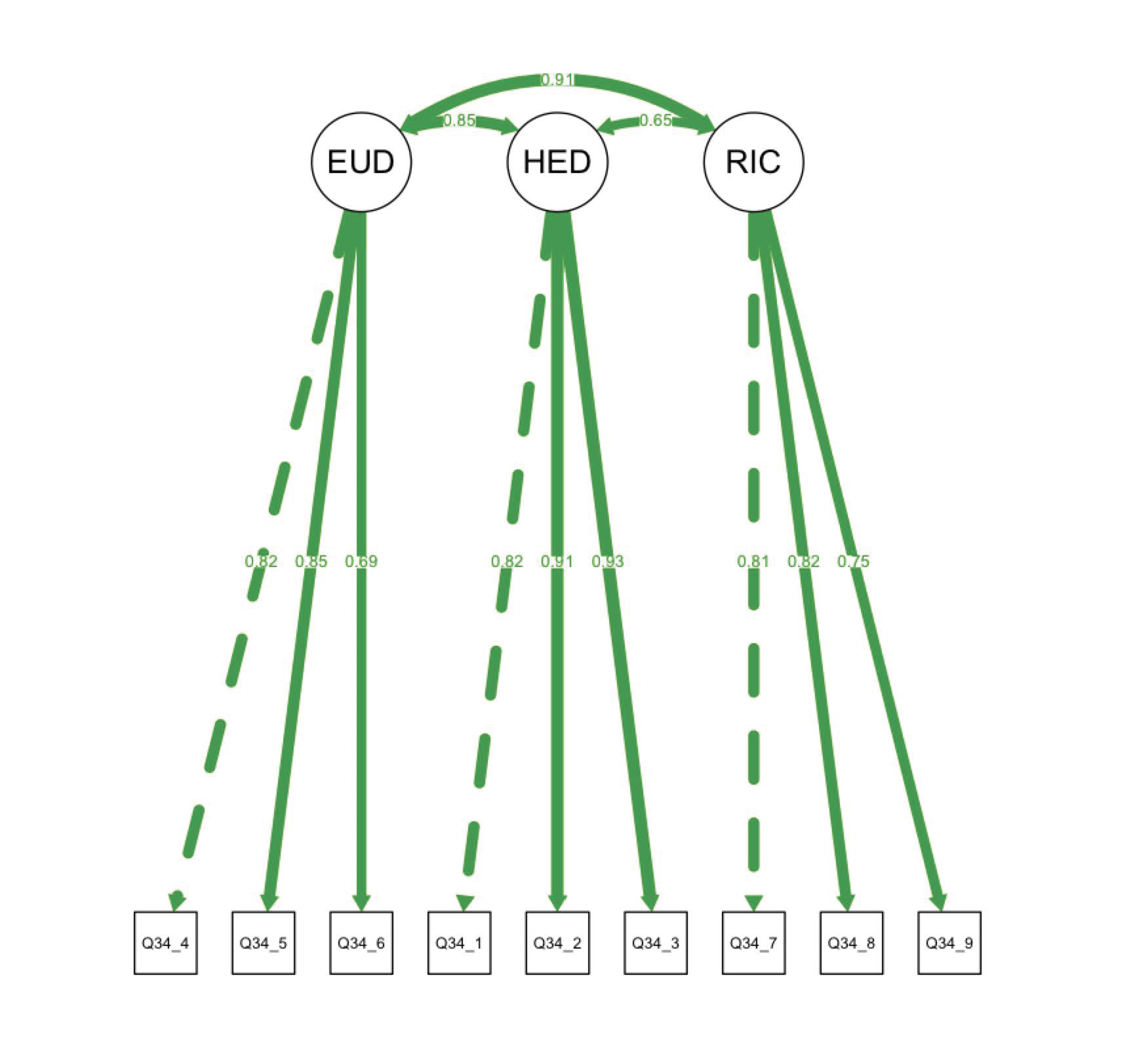

Supplement: S1 Fig — (DOCX) [file pone.0315596.s012.docx]

**S2 Fig. Factor loadings for the tripartite model of entertainment (study 2)**


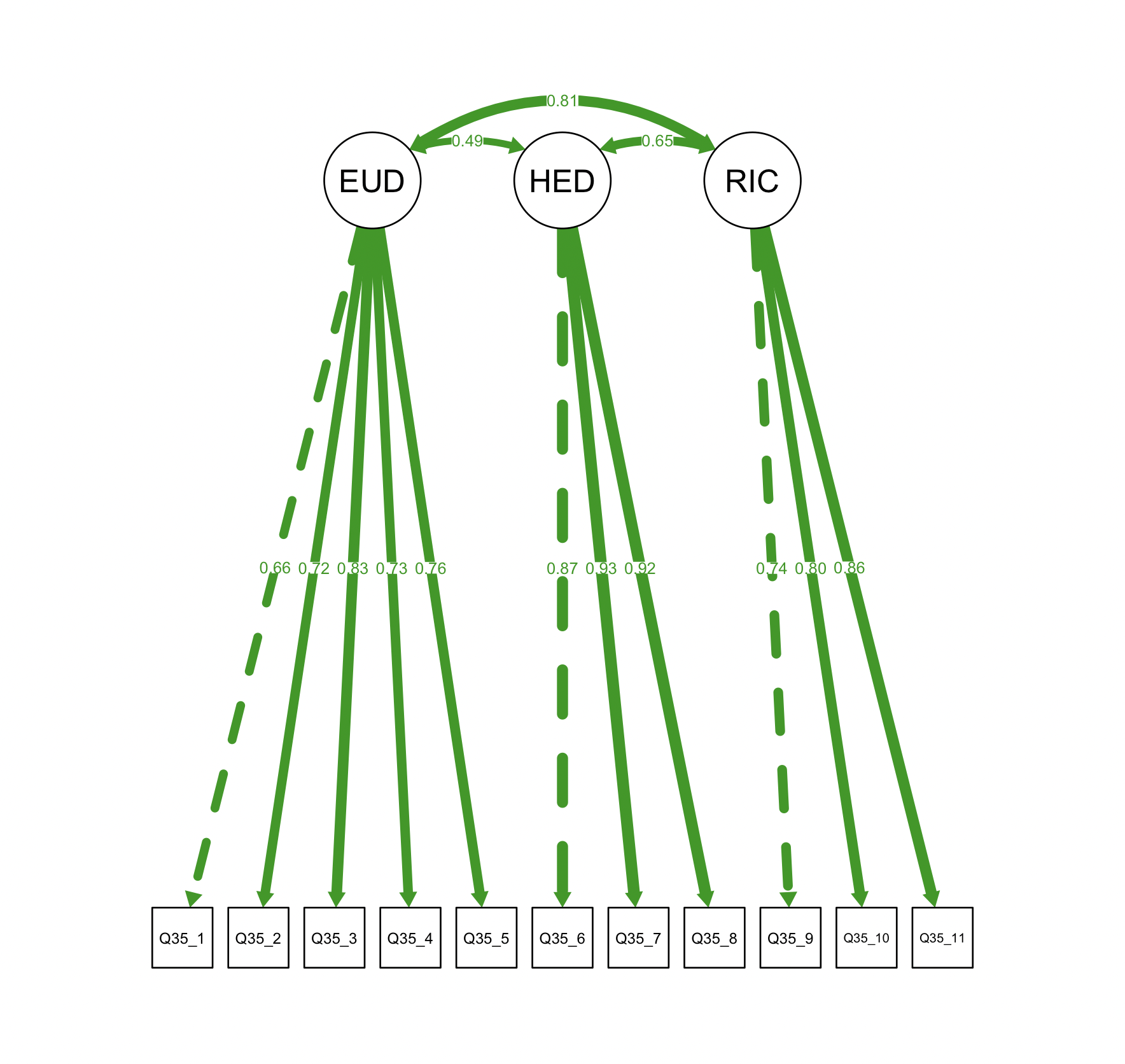

Supplement: S2 Fig — (DOCX) [file pone.0315596.s013.docx]

**S3 Fig. Factor loadings for the tripartite model of entertainment (study 3)**


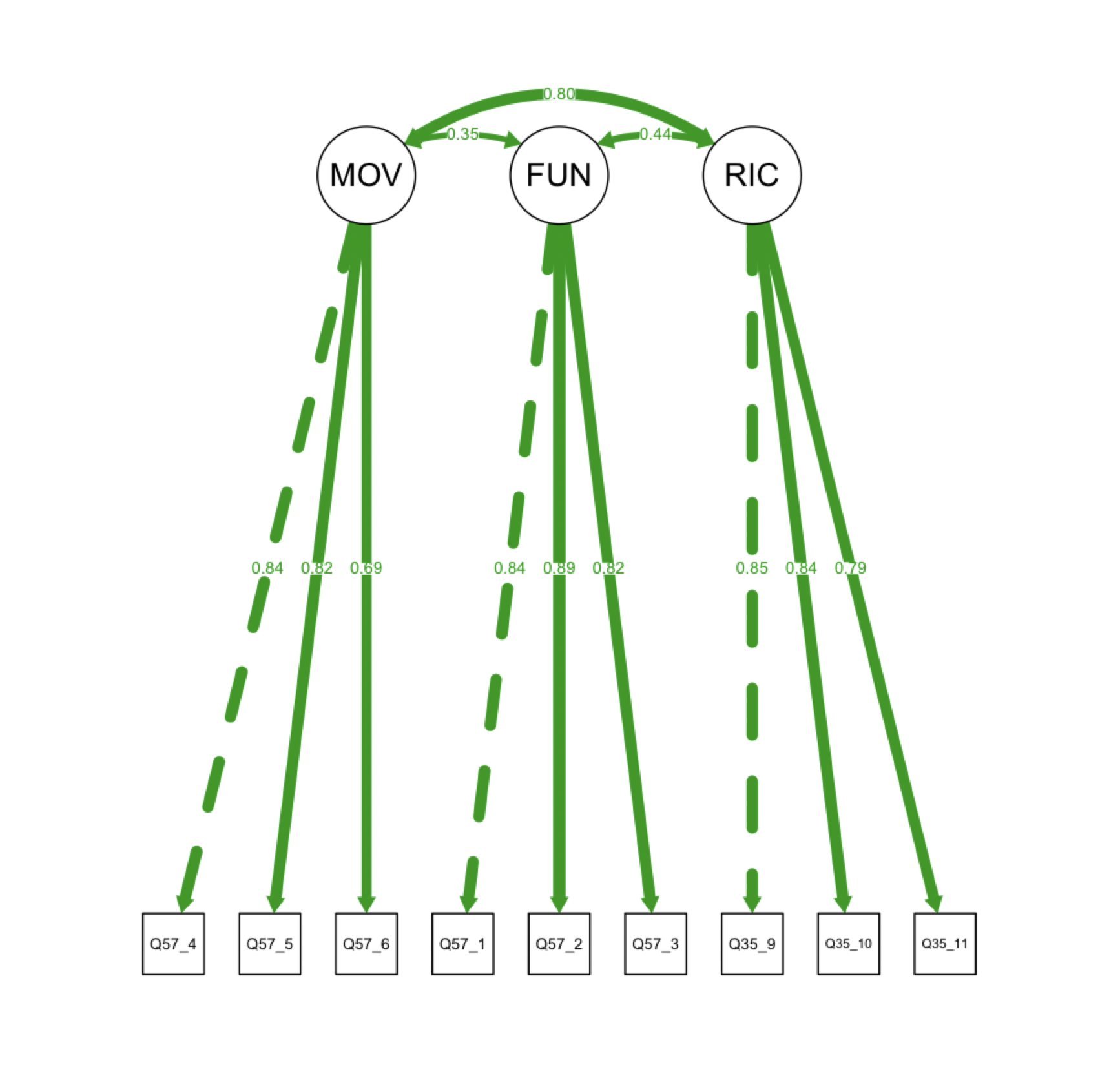

Supplement: S3 Fig — (DOCX) [file pone.0315596.s014.docx]
